# Genome‐Wide Association Study of Lean Body Mass Response to Resistance Training in Young Asians

**DOI:** 10.1002/jcsm.70347

**Published:** 2026-07-15

**Authors:** Zhuangzhuang Gu, Tao Mei, Xiaolin Yang, Zihong He, Jian Wu, Xiaoxia Li, Bing Yan, Yan Liang, Lijuan Liang, Jiayan Duan, Zhihao Zhang, Yaqi Wang, Xu Yan, Yanchun Li

**Affiliations:** ^1^ Henan Normal University Xinxiang Henan China; ^2^ Beijing Sport University Beijing China; ^3^ China Institute of Sport Science Beijing China; ^4^ Capital University of Physical Education and Sports Beijing China; ^5^ Shandong Sport University Jinan Shandong China; ^6^ Victoria University Melbourne Australia

**Keywords:** genetic predisposition score, genome‐wide association study, lean‐body mass, muscle hypertrophy, resistance training

## Abstract

**Background:**

Resistance training (RT) is an established intervention for increasing lean body mass (LBM), individual hypertrophic responses exhibit significant heterogeneity, largely influenced by genetic factors. This genome‐wide association study (GWAS) aimed to identify novel genetic variants associated with changes in lean body mass (∆LBM) following RT and to develop a genetic predisposition score (GPS) for predicting LBM trainability.

**Methods:**

A total of 187 sedentary adults (51.3% female, mean age 21.5 ± 2 years) completed a supervised 12‐week periodized RT programme. The protocol involved squats and bench presses at 70% 1‐repetition maximum (5 sets × 10 repetitions; 2‐min rest) performed twice weekly. ∆LBM was precisely quantified using dual‐energy X‐ray absorptiometry (DEXA) before and after the intervention. Genotyping was performed on venous blood‐derived DNA using the Illumina Global Screening Array‐24v1‐0. GWAS for ∆LBM was conducted using linear regression in PLINK 1.09, adjusted for baseline LBM, body mass index (BMI), age, region and sex (genome‐wide significance threshold: *p* < 5 × 10^−8^). A weighted GPS was subsequently constructed in SAS 9.3 based on significantly associated variants.

**Results:**

The RT programme elicited significant LBM gains across the cohort (*p* < 0.01) accompanied by high interindividual variability (∆LBM coefficient of variation = 0.84). GWAS identified nine novel single‐nucleotide polymorphisms (SNP) significantly associated with ∆LBM (*p* < 5 × 10^−8^): rs10212396, rs12519717, rs12055037, rs2131183, rs75968146, rs4370982, rs74038095, rs73966436, and rs12625907. The developed GPS explained 27.7% of the observed ∆LBM variance (*p* < 0.001). Bioinformatic annotation revealed that rs10212396 is located near the *ROBO2* locus—a gene potentially implicated in muscular adaptation, though the specific functional roles of most proximal genes identified remain to be fully characterized.

**Conclusions:**

This study, representing the first GWAS of RT‐induced LBM adaptation, successfully identified nine novel genetic variants and establishes a robust GPS that accounts for 27.7% of interindividual variation in LBM response. These findings provide foundational genetic markers crucial for deciphering the mechanisms underlying muscle hypertrophy and significantly advance the prospects for developing personalized exercise prescriptions.

## 1 Introduction

Bones and muscles are important components in humans, essential for movement and metabolism [1]. Increasing lean body mass is important for health, athletic performance and preventing chronic diseases such as diabetes, sarcopenia, and osteoporosis [2]. Physical activity has long been recommended to enhance fitness and health; in particular, ACSM recommended resistance training to enhance muscle strength, power and muscle hypertrophy [3].

The efficacy of resistance training for enhancing muscle strength and size in healthy adults is unequivocal. Persistent questions regarding the dose–response relationship—how variations in intensity, frequency, volume and exercise type differentially affect outcomes—continue to drive research in the field [4]. Beyond training variables, nutritional supplementation and dietary regimens are also recognized as critical factors modulating resistance training outcomes, particularly in older adults and populations with chronic diseases [5]. More pronounced training‐induced adaptations are typically observed in response to high‐intensity protocols, among novice trainees, and in younger participants [6]. A paradigm shift toward personalized physical activity prescription is gaining momentum in exercise science research, moving beyond one‐size‐fits‐all recommendations to individualized strategies based on personal characteristics. Understanding the factors that contribute to exercise response variation is the first step in achieving the goal of developing personalized exercise prescriptions [7]. Investigating the genetic factors underlying exercise sensitivity can provide new insights for developing personalized physical activity prescriptions [8].

Exercise genomics studies have revealed that not only sports performance but also trainability shows high genetic heterogeneity. Despite undergoing identical resistance training without nutritional supplementation, trained and novice individuals exhibit substantial variation in outcomes, and genetic factors are considered a major determinant. Previous studies with a hypotheses‐driven candidate gene approach have found that ACTN3, ACE, VDR, and so forth, gene polymorphisms are associated with the change in muscle hypertrophy following resistance training programme [9]. Most of these studies are from The Functional Single Nucleotide Polymorphisms Associated with Human Muscle Size and Strength (FAMuSS) project. However, except for ACTN3 and ACE genes, most of the candidate gene studies cannot be repeated [10]. With the development of sequencing technology, GWAS has been considered a powerful approach for genetic studies of complex traits as well as trainability. Guilherme [11] et al. have significantly advanced the field by identifying 57 genetic variants associated with muscle fibre size, which collectively contribute to a predisposition for power sports. Stands in contrast to the relatively limited genetic exploration dedicated specifically to resistance training‐induced muscle mass gains. A genome‐wide association study (GWAS) conducted in European‐ancestry populations on muscle hypertrophy following resistance training even found that none of the candidate genes can be repeated [12]. In the Asian context, a limited number of GWAS have begun to explore the genetic basis of resistance training‐related phenotypes—such as changes in muscle strength and lower limb muscle thickness [13, 14]. Genomic association data can be applied to the identification and prediction of athletic talent and training sensitivity [15].Muscle‐related phenotypes are complex and are polygenic (influenced by many genes working together). Both candidate gene studies and GWAS have revealed that with each genetic variant likely to contribute a small percentage (typically less than 1%) [16]. To address this issue, the method of genetic predisposition score (GPS) has been gradually introduced into exercise genomics [17]. Through the use of GPS, heritability studies have been able to show the role genetic factors play in the changes of muscular phenotypes after exercise intervention [18]. To the best of our knowledge, no studies have been performed combining GWAS with GPS to explain resistance training‐induced lean body mass changes in a healthy Asian population. Such studies might help us better understand individual adaptive variations toward exercise and can be useful for the design of individualized exercise prescriptions in the future.

In this study, a total of 187 Chinese Han young adults completed 12 weeks of resistance training; DNA was extracted from blood and genotyped using Infinium Chinese Genotyping Array v1.0 (Illumina Inc.). GWAS was used to identify the associations of genetic variations with the gains of LBM after 12 weeks of resistance training, and GPS was then used to predict the LBM trainability.

## 2 Methods

### 2.1 Participants

Participants were recruited from student cohort of Beijing Sport University, all participants provided informed consent and signed the consent form. Criteria for the enrollment of subjects: (a) Participants were defined as having an irregularly exercise lifestyle (i.e., less than 30 min per day and less than two times per week) based on the Global Physical Activity Questionnaire (GPAQ). (b) All participants were physically healthy with no major medical illnesses or current use of prescription medications. (c) Participants were not using anabolic agents (e.g., anabolic steroids, supplemental protein, creatine monohydrate or prohormones). This study was approved by the Ethics Committee of Beijing Sport University.

### 2.2 Training Regimens

The training regimens involved bench presses and squats: 10 sets of 8 repetitions, 70% of their one‐repetition maximum (1RM). Participants conducted two training sessions a week, separated by at least 2 days, for 12 weeks. The 1RM was also recalculated every 4 weeks to adjust the training load. Each training session started with 10–15 min of warm‐up; formal training was then carried out, and each group was supervised by one team member, and the attendance rate of the subjects was counted, requiring the subjects to complete all the intervention plan. All participants were instructed to maintain their current physical activity and dietary habits during the intervention period.

### 2.3 1RM Testing

Before the intervention, subjects completed the 1RM test. Test 1RM squat/bench press with a strength training rack, barbell bar and standardized bell pads. Test process was (1) estimate 1RM according to each subject's subjective feelings and perform five to 10 squats (bench press) at 40% of the estimated value as a warm‐up; (2) rest for 1 min after the warm‐up; (3) increase the weight by 15–20 kg on top of the warm‐up weight and allow the subject to complete 3–5 squats (bench press) as the first attempt; (4) after the completion of the first attempt, rest for 2–4 min; if the squat test is performed, increase the load from step 3 by 15–20 kg, and that the subject can complete about 2–3 squats at most; if the bench press test is performed, 5 to 10 kg is added to the load from Step 3, so that the subject can complete about 2 to 3 bench presses; (5) rest for 2–4 min, repeat Step 4, try 1RM; (6) if the subject successfully completes Step 5, continue to increase the load until the subject cannot successfully complete 1 squat (bench press) under the load; (7) rest for 2–4 min, reduce the load by 5–10 kg to continue the test, and then continue to increase or decrease the load until 1RM is achieved, and squat/bench press 1RM should be determined within five attempts.

### 2.4 LBM Measurement

LBM measurement was evaluated with dual‐energy X‐ray absorptiometry (DEXA [GE Healthcare, USA]). Before testing, subjects were required to fast for at least 8 h and abstain from barium meals, radioisotope injections or CT/MRI contrast agents within the preceding 7 days. During the test, subjects assumed a supine position after removing any clothing or items that could interfere with measurement. Whole‐body composition was assessed using the enCORE software (2011) by performing a sequential scan from head to feet, providing LBM data for the whole body.

### 2.5 DNA Isolation and Analyses

Five milliliters of venous blood was collected, and DNA was then extracted according to the Tiangen DNA extraction kit process. Ultraviolet spectrophotometer was used to detect DNA concentration and purity, and agarose gel electrophoresis was used to detect DNA purity and integrity. DNA was re‐extracted if the concentration did not meet the requirements or integrity was damaged. DNA was then stored at −80°C until used for the genotyping assay. Based on the high‐throughput genotyping platform, Bead Array with Infinium Chinese Genotyping Array v1.0 gene chip by Illumina (the total number of markers is 684 023) was used for gene scanning and genotyping. GenomeStudio 2.0 from Illumina was used to read the sequencing results and convert the data format.

### 2.6 Quality Control and Genotype Imputation

In pre‐imputation quality control (QC), SNP quality control procedures were conducted to eliminate ineligible SNPs (SNPs from mitochondria or X or Y chromosome, genotyping rate < 0.95, Hardy–Weinberg Equilibrium (HWE) *p*‐value < 10^−5^, and minor allele frequency (MAF) < 0.01), following which, 501 080 autosomal SNPs remained. SHAPE IT was used for phasing, and IMPUTE2 was used for imputation with imputation quality (*R* ^2^) > 0.8, HWE *p*‐value > 10–6, and SNP call rate > 0.97. Reference panels were from 1000 Genomes Phase 3 (v5). Plink (version 1.9) was used for QC of genotype data at both individual and SNP levels [19]. We controlled for high levels of missingness, indicative of poor DNA quality or technical problems (geno 0.1 and mind 0.1 for SNPs and individuals, respectively); we also set for minor allele frequency (‐‐maf 0.05). Lastly, we controlled for the Hardy–Weinberg equilibrium (HWE) rule (*p*‐value < 1.0 × 10^−5^), following which, 4 110 727 autosomal SNPs remained.

### 2.7 Statistical Analyses

Baseline characteristics of measurements were expressed in terms of mean ± standard deviation (SD). Association analysis was performed by using a linear regression additive model with region, sex, age, BMI and LBM baseline as covariates in PLINK v1.9, and statistical significance was established a priori at *p* < 1 × 10^−5^. The Manhattan and QQ plots were drawn using the R package qqman. The genomic inflation statistic (*λ*) of GWAS was calculated as the median of the resulting chi‐squared test statistics divided by the expected median of the chi‐squared distribution with one degree of freedom. Multiple stepwise regression analysis in *R* was utilized for the percent phenotypic variation explained (PVE) analysis. For post‐GWAS analysis: functional mapping and gene annotation (FUMA) was used as a main annotation tool for viewing and annotating GWAS results [20]. GPS was created using the Independent significant SNPs with the method of data‐driven GPS, which were calculated by summing the risk alleles for each independent SNP weighted from the GWAS study(0,1,2), and the contribution was calculated by stepwise regression using SAS 9.3.

## 3 Result

### 3.1 General Characteristics of Participants

A total of 187 (female 51.3%) subjects were enrolled. Participants' mean age was 21.5 ± 2 y, mean height was 1.71 ± 0.08 m, mean weight was 63.6 ± 13.1 kg, and mean body mass index (BMI) was 21.7 ± 3.2 kg/m2. After 12 weeks of training, LBM increased from 45.3 ± 9.9 kg to 47.0 ± 10.2 kg, with a net change of LBM by 1.7 ± 1.5 kg, and range from −3.6 to 7.6 kg. About 11% of subjects did not increase LBM, whereas 3% of subjects improved by 10% or more, and 86% of subjects between 0% and 10% (full information is listed on Table S1).

### 3.2 Genome‐Wide Association With △LBM—Percentage

**FIGURE 1:**
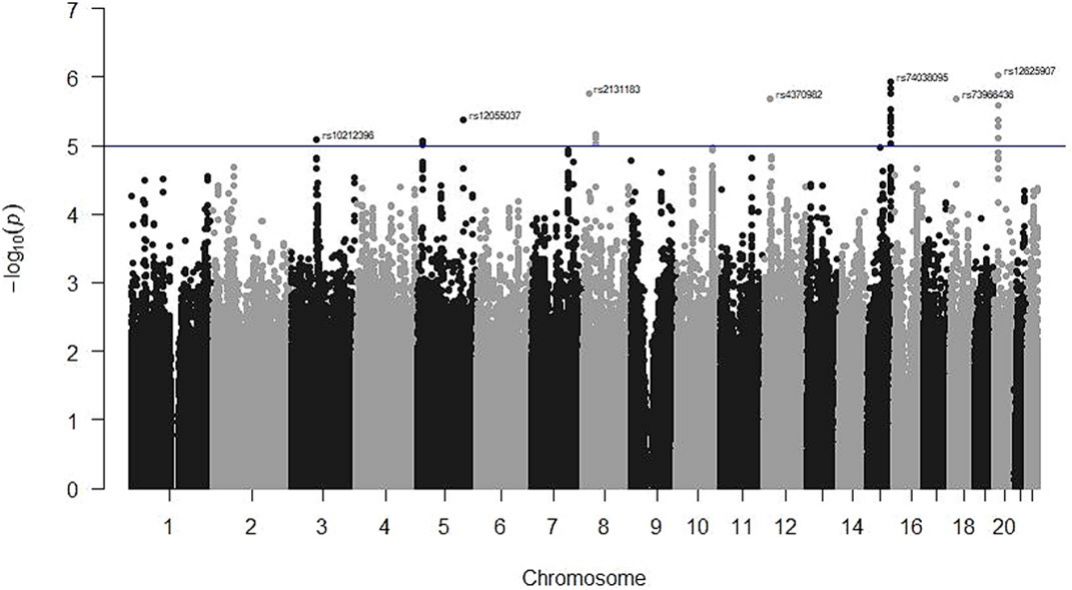
Manhattan plot and QQ‐plot of GWAS result. The plot shows the‐log10‐transformed *p*‐values for all SNPs.

The GWAS results are summarized in the Manhattan plot (Figure 1). The quantile‐quantile (Q‐Q) plot (Figure S1) indicated a genomic inflation factor (*λ*) of 1.014, suggesting minimal influence from population structure or other confounding factors. Our GWAS indicated that 44 SNPs were associated with LBM training response at the *p* < 1 × 10^−5^ significance level. Nine significant independent SNPs were identified at the *r* ^2^ < 0.8. SNP with the strongest association is rs12625907 (*β* = 2.306, minor allele frequency = 0.22, PVE = 3.67%, *p* = 9.28 × 10^−7^), which is located on the intergenic of the SPTLC3A and ISM1 gene, closer to ISM1. SNP with the highest PVE is rs10212396 that can account for 7.57% individual differences among △LBM (*β* = −1.641, minor allele frequency = 0.27, PVE = 7.59%, *p* = 8.20 × 10^−6^), which is located on the intronic region of the ROBO2 gene. Full results of significant independent SNP analyses are given in Table 1, and associated SNPs are listed in Table S2.

**TABLE 1:** The results of Genome‐wide association with △LBM—percentage and functional annotations for associated SNPs.

rsID	Chr	pos	EA	NEA	MAF	Beta	PVE(%)	CADD	RDB	Gene/nearest gene	Func	*p*
rs10212396	3	77 476 889	C	T	0.2748	−1.641	7.59	20.7	2a	ROBO2	Intronic	8.20*10^−6^
rs12519717	5	16 984 504	A	G	0.1577	2.332	6.97	1.675	5	RNU6‐660P	Intergenic	8.71 *10^−6^
rs12055037	5	141 815 823	G	A	0.1121	2.823	6.77	0.072	6	AC005592.2	ncRNA_intronic	4.27 *10^−6^
rs2131183	8	18 664 444	C	G	0.1944	2.177	3.99	0.925	7	PSD3	Intronic	1.71 *10^−6^
rs75968146	8	39 244 466	A	C	0.07738	3.039	4.21	6.136	7	ADAM5	ncRNA_intronic	6.71 *10^−6^
rs4370982	12	20 988 657	C	G	0.08433	3.408	2.98	5.331	7	SLCO1B3	Intronic	2.10 *10^−6^
rs74038095	15	91 715 408	A	C	0.05952	3.447	1.53	3.776	5	SV2B	Intronic	1.18 *10^−6^
rs73966436	18	20 635 776	C	T	0.05655	3.543	5.71	0.058	5	RBBP8	Intergenic	2.06 *10^−6^
rs12625907	20	13 163 445	A	G	0.2183	2.306	3.67	0.175	6	SPTLC3, ISM1	Intergenic	9.28 *10^−7^

*Note:* EA: effect‐allele; NEA: no‐effect‐allele; MAF: minor allele frequency; PVE: percent phenotypic variation explained (Partial *R* ^2^); CADD, CADD score, which is computed based on 63 annotations. The higher the score, the more deleterious the SNP is. 12.37 is the suggested threshold; RDB, Regulome DB score, which is a categorical score (from 1a to 7, 2a: TF binding + matched TF motif + matched DNase Footprint + DNase peak; 5: TF binding or DNase peak; 6: Motif hit; 7: Other).

According to the CADD score and RDB score, the SNP most likely to influence gene expression is rs10212396, which is likely to change the expression of ROBO2. ROBO2 gene plays an important role in nervous system development and regulation of expression of SLITs and ROBOs (full information is listed in Table S2). Utilizing the GTEx Portal database, we analysed a possible association of all SNPs with altered gene expression in various tissues, and we found that rs12519717 and its linkage SNP may influence MYO10 gene expression in Thyroid; rs75968146 may influence HTRA4 gene expression in Adipose Visceral Omentum and PSD3 gene expression in Brain Spinal cord cervical; rs74038095 and its linkage SNP may influence SV2B gene expression in many connective and nerve tissues such as Adipose, Colon_Sigmoid, Skeletal Muscle, Nerve_Tibial and Pancreas tissues; rs12625907 may influence ISM1 gene expression in Thyroid (full information is listed on Table S3).

### 3.3 Genetic Prediction Scores

**FIGURE 2:**
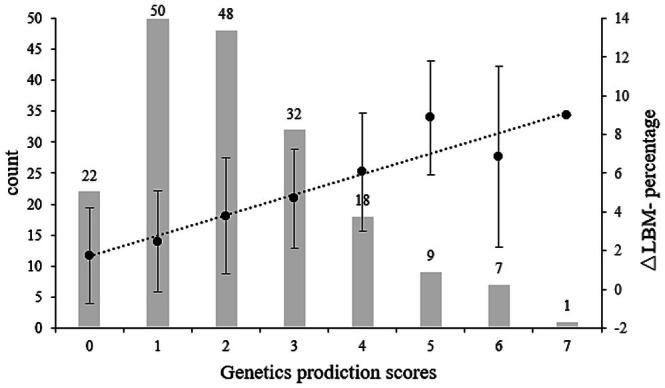
Distribution of GPS and its linear regression model with △LBM—percentage after 12‐week RE intervention. The bar graphs show distributions of GPS, and the line chart indicates the △LBM—percentage.

According to the GWAS results, nine SNP were assigned and added to calculate GPS. The maximum value of GPS is 7 and the minimum value is 0. The distribution of PGS is shown in Figure 2. GPS was positively correlated with △LBM in response to resistance training, whereas BMI was negatively correlated with the resistance training effect in △LBM according to the stepwise regression analysis with part, age, gender, BMI, and LBM baseline as covariates. The higher the GPS score, the higher the LBM growth rate after 12 weeks of resistance training, and the GPS could explain 27.7% of the variance in △LBM percentage (Table S4 and Figure 2).

## 4 Discussion

### 4.1 Main Findings of This Study

This manuscript described the GWAS of resistance training response among the young Chinese Han population. In this study, we identified nine novel SNPs (rs10212396, rs12519717, rs12055037, rs2131183, rs75968146, rs4370982, rs74038095, rs73966436 and rs12625907) that might differentiate the resistance training‐induced changes in LBM. To our knowledge, most of the sports genomics studies focus on Caucasians, and different ethnic groups often get different results because of differences in genetics and living environment [21]. These findings contribute to a broader understanding of the genetic underpinnings of muscle plasticity by providing evidence of its relevance across diverse ethnic groups. The GPS calculated by nine SNPs explained 27.7% of the individual differences of △LBM after 12 weeks RE. The higher the GPS score, the more significant the muscle‐building effect achieved during resistance training. These results may provide a reference for personalized exercise prescriptions.

### 4.2 Potential Genetical Mechanism for Resistance Training to Promote LBM

In our study, we speculate that the nervous system development may be associated with resistance training‐induced LBM gains. The majority of complex trait associated variants identified from GWAS reside in noncoding DNA sequences, and it is difficult to determine the function of those SNPs. It is noteworthy that all nine associated SNPs are situated in noncoding regions, and none replicate the findings from prior exercise genomics research. This underscores the population‐specific or phenotype‐unique nature of our discoveries and emphasizes the need for future functional work to decipher their biological mechanisms [13, 14]. This observation further supports the nature of complex polygenic traits and the fact that the majority of trait‐associated SNPs discovered by GWAS reside in noncoding regions of the genome. These noncoding variants are believed to influence gene regulation rather than protein structure. A key challenge for future research will be to elucidate the functional mechanisms of these noncoding SNPs [22]. In this current study, we mainly used FUMA to explore the function of SNPs with significant difference. According to the CADD score and RDB score, only rs10212396 is most likely located in a functional region and is the causal variant; other SNPs are less likely located in a functional region based on the current evidence. The closest gene of rs10212396 is ROBO2, which is a main receptor for SLIT2 and probably SLIT1, and ROBO2 functions as a SLIT3 receptor to aid myoblast differentiation [23]. They are thought to act as molecular mediators in cellular migration, including axonal navigation at the ventral midline of the neural tube and projection of axons to different regions during neuronal development. It was reported an association between ROBO2 polymorphism and grip strength based on data from UK biobank [24].

Resistance exercise is used extensively in athletic and general populations to induce neuromuscular adaptations to increase muscle size and performance. Neurological factors may make their greatest contribution during the early stages of a resistance training programme [25]. Loss of nervous system modulation disrupts muscle homeostasis and leads to changes in the expression of muscle‐related genes. We speculate that ROBO2 plays an essential role in the morphogenesis of the nervous system, especially the peripheral nervous system [26], leading to different resistance training responses. There is currently no direct evidence to suggest that ROBO2 is related to muscle hypertrophy response to resistance training, which requires further investigation. Besides, from the GTEx database, we also found this SNPs may influence the expression of multiple genes (MYO10, HTRA4, PSD3, SV2B and ISM1) in a variety of tissues. Among them, MYO10 is also associated with the development of the nervous system [27], and a study also reported that MYO10 polymorphism was associated with sarcopenia among Koreans. Similar to the ROBO2 gene, there is also a lack of adequate evidence to support the relationship of this gene with the muscle hypertrophy response to resistance training. Future studies are required to explore the functional significance of this SNP, such as knockdown studies to verify their roles in the muscle hypertrophy response to resistance training. Elucidating the molecular mechanisms underlying exercise adaptability has long been a central focus in sports science [28]. Our findings provide the first evidence from a GWAS for nine novel SNPs associated with the change in lean body mass following standardized resistance training in a young Han Chinese cohort. By characterizing the dynamic response to standardized training rather than static traits, our findings directly address the genetic architecture of trainability.

### 4.3 GPS Model as a Prediction Tool of Resistance Training Response

It is known that a single SNP can only explain a small percentage of individual variances for training responses. In our study, a single SNP could only explain 1.53% to 7.59% of the individual differences of LBM gains in the response to RE. Our results are consistent with some candidate gene studies. Pescatello et al. [16] reported that 17 genes were associated with muscle strength or size gains after 12 weeks RE from the FAMuSS cohort, and the contribution of a single SNP is between 1% and 4%. Because of the small contribution of a single SNP, it is almost impossible to use a single SNP to predict the training effect in practical applications. There is a necessary need for a method to combine the influences of multiple SNPs. In early studies, different SNPs in the same gene can construct haplotypes to improve the interpretation. When there are more genes associated with the phenotype, polygenic risk scores (PRS) were used to predict the influences of genetic variants to diseases in the medical field [29]. In the field of sports genomics, total genotype score (TGS) and GPS have been used to predict sports performance and training response [17, 18]. In this study, to better predict the muscle gaining effect of resistance training, we used the data‐driven GPS method. Another study reported by He et al. [30] found that data‐driven GPS based on nine SNPs explained 27% of the variance in ∆SMM following 1 year of RE among older people. The results were very similar to ours, and we found our GPS could explain 27.7% individual variances of △LBM in response to RE. Intriguingly, SNPs we used were different from the study by He et al. More SNPs may further improve the interpretation; for example, Zillikens et al. [31] reported that enough SNPs might explain 43.3%–44.2% of the lean body mass variance. Therefore, more SNPs may be added to build the GPS model in future studies.

Human skeletal muscle mass reaches its peak at 25 years old and then declines by 3%–7% every 12 years. Regular resistance training in young adults can improve muscle mass and slow down ageing‐related decline in muscle mass, thus effectively preventing the occurrence of diseases such as sarcopenia and osteoporosis [32]. This study suggested that after 12 weeks of resistance training, some subjects' LBM did not increase, but rather decreased, similar results were also reported from the FAMUSS cohort [16]. There is an inverted U‐shaped trend between the intensity of resistance training, plus the amount of training, and the training effect. To ensure subjects achieve better muscle gaining effects in resistance training, it is necessary to design a more precise and personalized resistance training programme [33]. In the field of sports genomics, it is generally believed that the synergistic effect of environmental factors and genetic factors affects training sensitivity [15, 34]. In this study, we observed a negative correlation between baseline BMI and the increase in LBM. Furthermore, none of the nine identified SNPs showed a significant association with baseline BMI. When we included baseline BMI as a covariate in our GWAS model, the associations between these SNPs and ΔLBM remained significant. This indicates that the relationship between BMI and resistance training‐induced muscle hypertrophy is independent of the genetic effects captured by our SNPs. However, the precise mechanistic role of BMI in modulating the response to resistance training warrants further investigation. Similar to BMI, environmental factors are complex or even uncontrollable in some cases, but genetic factors are relatively stable. A better understanding of genetic factors may reveal individual training sensitivity, and genetic information can be used in the prediction of resistance training response [35]. Our finding provides novel, population‐specific genetic insights. The nine SNPs constituting our GPS are distinct from previously reported variants in other ethnic groups, underscoring the importance of expanding genetic studies to diverse populations. This finding challenges the universality of existing candidate genes and emphasizes that the genetic architecture of training response may be population‐dependent.

### 4.4 Limitations

Firstly, the sample size of this study was small, whereas consistent with some preliminary exercise genomics studies [12, 36], it falls short of the standards recommended for well‐powered GWAS [15]. Second, genotyping was conducted using chip‐based assays; we saved the DNA samples from the subjects and are planning to conduct whole‐genome sequencing in the future [37]. Third, our study did not validate results across independent cohorts. Finally, SNP function prediction has been carried out by FUMA; these results are still lacking experimental verification. Therefore, the results of this study need further validation.

### 4.5 Future Directions

Large‐scale, multi‐ethnic GWAS and whole‐genome sequencing studies are warranted to identify a more comprehensive set of genetic determinants of resistance training response, including rare variants, copy number variation (CNV) and so forth [38]. The functional impact of the prioritized SNPs, particularly those in noncoding regions, must be experimentally characterized. Techniques such as real time RT‐PCR (qRT‐PCR), CRISPR‐Cas9 genome editing in cell models and expression quantitative trait locus (eQTL) analysis are critical to link genetic associations to molecular mechanisms [39]. In clinical translation and personalization field, future work should focus on integrating the GPS with other omics data (e.g., transcriptomics, proteomics) and environmental factors to build more powerful predictive models [7]. Subsequently, randomized controlled trials are needed to test whether using these integrated models to guide personalized exercise prescriptions leads to superior health and performance outcomes.

## 5 Conclusions

This is the first GWAS of RT‐induced LBM adaptation, successfully identified nine novel genetic variants and establishes a robust GPS that accounts for 27.7% of interindividual variation in LBM response. These findings provide foundational genetic markers crucial for deciphering the mechanisms underlying muscle hypertrophy and significantly advance the prospects for developing personalized exercise prescriptions.

## Conflicts of Interest

Zhuangzhuang Gu, Tao Mei, Xiaolin Yang, Zihong He, Jian Wu, Xiaoxia Li, Bing Yan, Yan Liang, Lijuan Liang, Jiayan Duan, Zhihao Zhang, Yaqi Wang, Xu Yan and Yanchun Li declare that they have no conflicts of interest.

## Supporting information


**Figure S1:** Supporting information.

**Table S1:** General characteristics of participants.
**Table S2:** All SNPs in LD (*r* ^2^ > 0.6) of identified independent significant SNPs.
**Table S3:** Abbreviations and full names, pathway and Gene Ontology (GO) annotations of the nearest Gene of the SNPs.
**Table S4:** Significant single‐tissue eQTLs for loci associated with resistance training response in all tissues.
**Table S5:** Results of the stepwise regression analyses.
