# Supplementary figures and images for: Genome‐Wide Association Study of Lean Body Mass Response to Resistance Training in Young Asians
Source: J Cachexia Sarcopenia Muscle. 2026 Jul 15;17(4):e70347. doi: 10.1002/jcsm.70347 (PMC13371584; doi:10.1002/jcsm.70347)

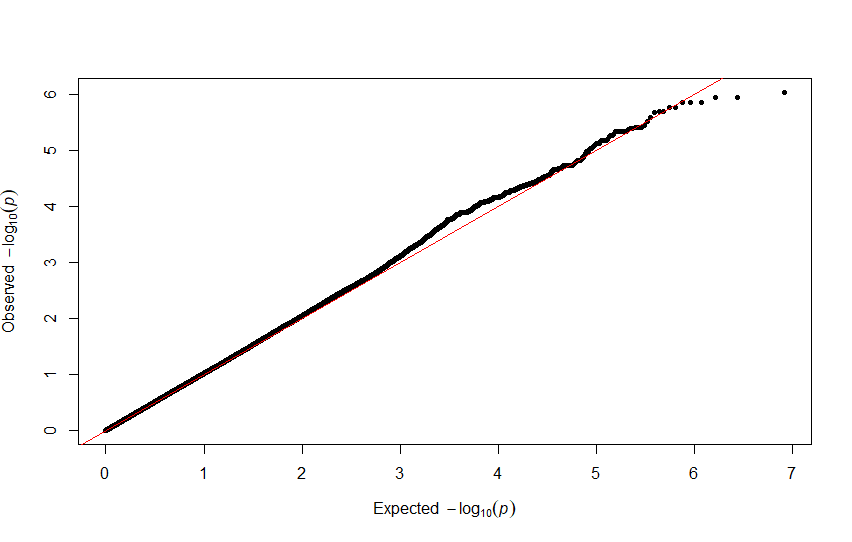

Supplement: Supplementary file 1 — Figure S1: Supporting information. [file JCSM-17-e70347-s001.bmp]
